# Mutant p53 Attenuates the Anti-Tumorigenic Activity of Fibroblasts-Secreted Interferon Beta

**DOI:** 10.1371/journal.pone.0061353

**Published:** 2013-04-22

**Authors:** Shalom Madar, Einav Harel, Ido Goldstein, Yan Stein, Ira Kogan-Sakin, Iris Kamer, Hilla Solomon, Elya Dekel, Perry Tal, Naomi Goldfinger, Gilgi Friedlander, Varda Rotter

**Affiliations:** 1 Department of Molecular Cell Biology, Weizmann Institute of Science, Rehovot, Israel; 2 Faculty of Biochemistry, Biological Services Unit, Weizmann Institute of Science, Rehovot, Israel; Bauer Research Foundation, United States of America

## Abstract

Mutations in the p53 tumor suppressor protein are highly frequent in tumors and often endow cells with tumorigenic capacities. We sought to examine a possible role for mutant p53 in the cross-talk between cancer cells and their surrounding stroma, which is a crucial factor affecting tumor outcome. Here we present a novel model which enables individual monitoring of the response of cancer cells and stromal cells (fibroblasts) to co-culturing. We found that fibroblasts elicit the interferon beta (IFNβ) pathway when in contact with cancer cells, thereby inhibiting their migration. Mutant p53 in the tumor was able to alleviate this response via SOCS1 mediated inhibition of STAT1 phosphorylation. IFNβ on the other hand, reduced mutant p53 RNA levels by restricting its RNA stabilizer, WIG1. These data underscore mutant p53 oncogenic properties in the context of the tumor microenvironment and suggest that mutant p53 positive cancer patients might benefit from IFNβ treatment.

## Introduction

The tumor microenvironment has gone well into the mainstream of cancer research, manifested by a constant flow of publications and by a growing interest coming from anti-cancer drug companies. It was even professed as a novel ‘hallmark’ of cancer [1], [2], [3]. Cancer Associated Fibroblasts (CAFs) - a sub population of stromal cells residing adjacently to the tumor, are considered pro-tumorigenic, and in some cancers serve as prognostic markers for the course of the disease [4]. CAFs exhibit several distinct features compared to normal fibroblasts including rapid proliferation rate, enhanced production of collagens, secretion of growth factors and other extra cellular modulators, and activation of unique expression programs [5], [6], [7], [8], [9], [10].

p53, a well-known tumor suppressor [11], is frequently mutated in tumors resulting in the expression of tumor promoting mutant forms. Several studies have addressed the role of mutant p53 in the tumor-stroma interaction [12]. For example, mutant p53 expressed in stromal cells surrounding prostate tumors, enhances tumor growth and facilitates metastasis [13]. In addition, a clear correlation was revealed between mutant p53 and VEGF expression, and tumor aggressiveness [14], [15]. Moreover, mutant p53 was reported to cooperate with E2F to induce the expression of ID4, which in turn leads to augmented angiogenesis [16].

Interferons (IFNs) are a group of cytokines that serve as a defense mechanism against viral infections and have the capacity to affect the transformation process. There are two major types of interferons – type I IFNs, mainly represented by IFNα and IFNβ, and type II IFNs, represented by IFNγ. Type I IFNs are produced by all nucleated cells, they bind a cell surface receptor encoded by IFNAR1/2 and can potentially initiate four different pathways. The canonical pathway includes the activation of JAK1 and TYK1, which relays the signal onto STAT1/2 by phosphorylation. STAT1/2 form a complex with IRF9 that translocates to the nucleus, where it binds IFN-stimulated response elements (ISRE) residing in the promoters of IFN target genes [17]. IFNβ seems to have a pleiotropic effect on cancer. On the one hand, IFNβ directly inhibits tumor growth when secreted by the tumor microenvironment [18]. On the other hand, IFNβ partakes in tumor escape from the immune system, either by selecting for IFN non-responsive cells [19] or by contributing to oncogenic Ras transformation [20] and enriching for cancer initiating cells [21]. Although IFNβ seems to cooperate with wild type p53 in tumor suppression and stress responses [22], [23], [24], its interaction with the mutant forms of p53 has not been investigated. In addition, the cross-talk which takes place between cancer cells expressing mutant p53 and CAFs is under-studied. When characterizing this interaction we revealed that CAFs induce IFNβ pathway in response to the presence of cancer cells - a response which was accentuated when the cancer cells expressed mutant p53 forms. Furthermore, CAFs-induced IFNβ response was moderated by mutant p53 via SOCS1 mediated inhibition of STAT1 phosphorylation. IFNβ on the other hand, reduced mutant p53 RNA levels by down regulating its RNA stabilizer WIG1. These results underscore the significance of characterizing p53 mutations in cancer, and imply that IFNβ treatment might prove to be beneficial for mutant p53 carrying patients.

## Results

### Establishment of an in vitro model to study the tumor-stroma encounter in lung cancer

**Figure 1:**
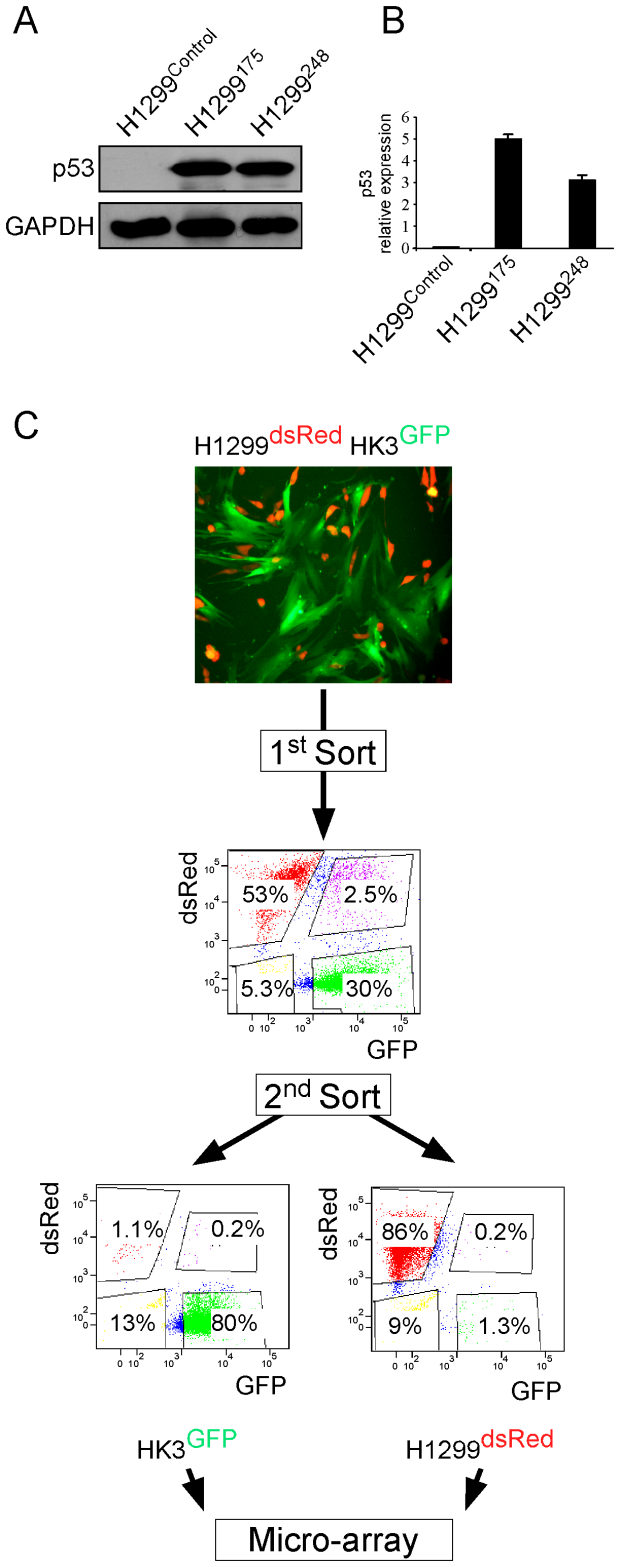
An in vitro model to study the tumor-stroma encounter in lung cancer. (A). p53-null lung carcinoma cells (H1299) were introduced with the designated mutations. p53 levels were determined by Western blot analysis (A) and by QRT-PCR (B). A fluorescent microscope image of co-cultured dsRed-labeled H1299 with GFP-labeled HK3 (C, upper panel). Representative FACS analysis depicting dsRed- and GFP-labeled sub-populations following a sorting procedure (C, middle panel). Each sub-population was then re-sorted using the same sorting gates (C, lower panel).

As stromal cells often reside in, or are recruited to the vicinity of the tumor, we sought to establish an in vitro co-cultivation model that recapitulates this encounter and permits an efficient separation and characterization of the two cell populations. As we planned to investigate the effect of mutant p53, we chose to work with lung cancer cells (H1299) which are null for p53 expression and introduced them with two p53 ‘hotsopt’ mutations residing within the DNA binding domain, namely R175H and R248Q (H1299^175^ and H1299^248^ respectively, Figure 1A and B). The cells were then labeled with a red fluorescent protein (dsRed), while lung CAFs (HK3-T) were labeled with a green fluorescent protein (GFP). The labeled populations were co-cultivated for 24 hours and separated by Fluorescence Associated Cell Sorting (FACS) based on their specific fluorescent marker (Figure 1C). To minimize the possibility of cross contamination, the separated populations were sorted again, and indeed, the level of cross contamination was diminished (Figure 1C). To further corroborate this observation, we also performed quantitative real time PCR (QRT-PCR) with primers amplifying either GFP or dsRed. Following the double-sorting procedure, GFP and dsRed expression was several orders of magnitude higher in the corresponding labeled cells (Figure S1A). Because the sorting procedure includes prolonged incubation on ice, and cells may be subjected to mechanical stress introduced by the FACS machinery, we decided to measure the expression levels of stress-related genes prior and post the sorting procedure. First, p21, a common stress response gene, was found to be expressed in a comparable manner in the sorted and unsorted samples (Figure S1B). Moreover, several other genes that are known to be specifically elevated during mechanical stress in lung cells [25] were found to be either equally expressed or down regulated following the sorting procedure (Figure S1B).

Taken together, these results indicate that our experimental system is capable of separating the two cell populations with a high degree of purity, without imposing measurable mechanical stress.

### CAFs invoke the interferon beta pathway in response to the presence of cancer cells

**Figure 2:**
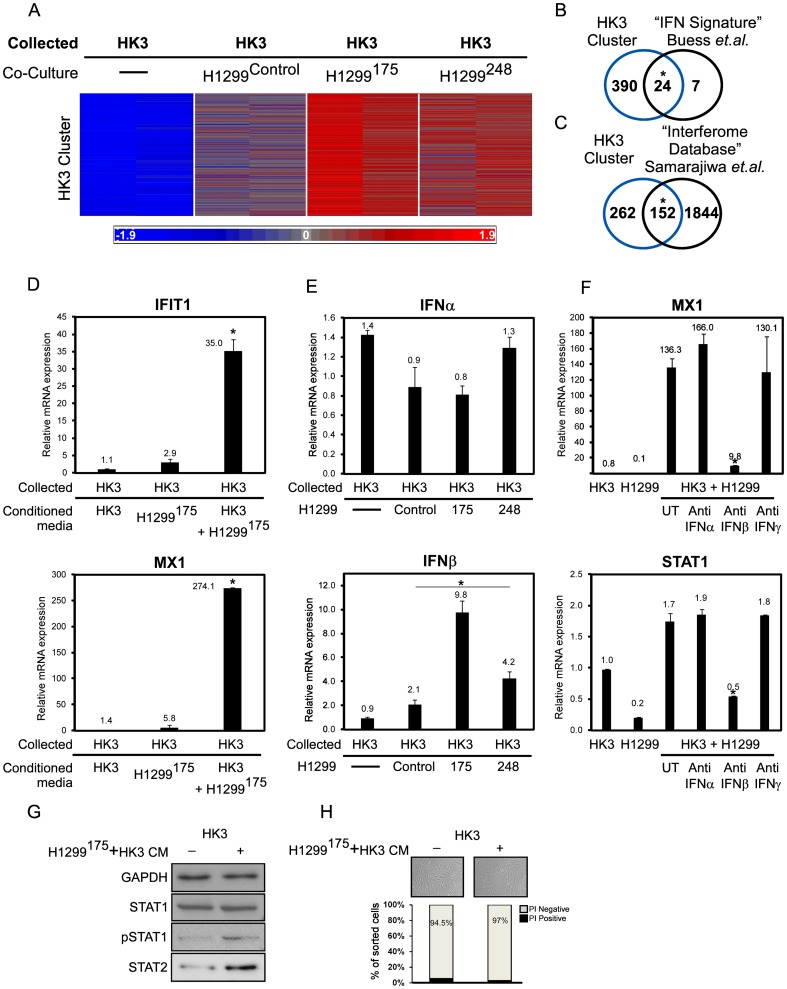
The interferon beta pathway is up regulated in fibroblasts after co-cultivation with mutp53-bearing carcinoma cells. (A) Following the described sorting procedure, HK3-T samples were subjected to a microarray analysis (see materials and methods). The presented cluster was obtained by the ‘CLICK’ algorithm from the ‘Expander’ package using default homogeneity (version 5.2) [27]. The log 2 ratios were standardized to have zero mean and unit standard deviation for each gene. (B) ‘HK3-T cluster’ which contains 414 genes was compared to a previously reported ‘IFN signature’ that was induced by co-cultures of cancer and stromal cells. (C) ‘HK3-T cluster’ was compared with the ‘Interferome database’ containing 1196 IFN targets. A Fisher's exact test was utilized to compare these overlaps with those of all other clusters. * = P<0.0001 (D) To assess the significance of the physical interaction between HK3-T and H1299^175^ in eliciting the IFN pathway, HK3-T were introduced to conditioned media from each cell type grown alone or from a co-culture plate. mRNA levels of two known IFN targets were measured by QRT-PCR. (E) Interferon α and β mRNA levels. (F) CAFs and H1299 were grown either alone or in a co-culture. The co-cultured cells were incubated with the designated antibodies. Shown is the mRNA level of the designated IFN targets. * P<0.05. (G) HK3-T cells were subjected to conditioned media of HK3-T or that of HK3-T cultured with H1299^175^. Shown is a western blot of GAPDH, STAT1, pSTAT1 and STAT2. (H) The same experimental setup was used. Shown in the upper panel are microscope images of the cells. Cells were then collected and stained with Propidium Iodide (PI) and apoptotic cells (PI positive) were detected by FACS sorting and their percentage is depicted in the lower panel.

To gain insights into the gene expression profile of stromal cells following the encounter with mutant p53 expressing cancer cells, we analysed the differentially expressed genes (two fold change or more, 0.05 p-value or less) in HK3-T before and after co-cultivation with either p53 null, H1299^175^ or H1299^248^ cancer cells via micro-array. This comparison yielded a list of 875 differentially expressed genes that were clustered into 8 distinct groups by the CLICK algorithm using the Expander package (version 5.2) [26], [27]. Of note, is the first cluster (‘HK3-T cluster’, Figure 2A) composed of a group of 414 genes induced by the mere co-cultivation with carcinoma cells. This induction was further enhanced in the presence of mutant p53 expressing cells. The ‘HK3-T cluster’ was further characterized by the use of IPA algorithm (Ingenuity® Systems) [28] which identifies enriched Gene Ontology annotations and canonical pathways within a given list of genes. The most significantly enriched term was the “interferon signalling pathway”, for which 14 genes out of 36 were elevated in HK3-T (p-value – 8.4×10^−16^) in response to co-cultivation with carcinoma cells. Furthermore, in a study by Buess and colleagues, breast stromal cells and breast cancer cell lines were co-cultivated and subjected to micro-array analysis, and the most significant cluster was enriched with an interferon signature consisting of 31 genes [29]. This ‘interferon cluster’ was compared with the ‘HK3-T cluster’ and yielded an overlap of 24 out of 31 genes (Figure 2B). Moreover, the ‘HK3-T cluster’ was compared with a database of ∼2000 known interferons targets termed ‘interferome’ [30] and an enrichment of 37% (152 out of 414) was observed (Figure 2C). Buess et. al. also reported that a cell-cell interaction between cancer and stromal cells is required to induce IFN response in the former [29]. Accordingly, we set out to determine whether physical interaction between the cells is a prerequisite for triggering the IFN pathway or whether carcinoma cells grown alone are able to secrete factors, which evoke such a response without the presence of CAFs. Conditioned media collected from carcinoma cells grown alone induced a slight elevation of IFN targets in CAFs (Figure 2D). A more prominent effect however, was observed when conditioned media collected from a co-culture of CAFs and carcinoma cells was transferred to the CAFs.

To rule out the possibility of cell line specific effects, we decided to compare several other combinations of CAFs and lung carcinoma cells. As illustrated in Figure S2A–C, HK3 were able to induce the IFN pathway when co-cultivated with the carcinoma cell line A549, but not with H460. Moreover, CAFs derived from another patient were able to evoke the IFN pathway as well, however, not when co-cultured with each other (Data not shown). These data suggest that the interferon pathway is up regulated in some but not all pairs of CAFs and lung carcinoma cells and not in the presence of normal cells. All interferons share mutual targets, and more specifically type I interferons (α and β) are almost inextricable with regards to their targets, and mainly differ by their affinity to the type I interferon receptors [31]. To differentiate between the interferons and reveal the identity of the predominant cytokine in our experimental model, we compared the mRNA expression levels of interferons α, β and γ. Interferon γ was not detected in CAFs, regardless of the presence of carcinoma cells. Interferon α levels were comparable between the samples, whereas interferon β levels were elevated in CAFs when cultivated with carcinoma cells. In the presence of carcinoma cells expressing mutant p53, IFNβ levels were further induced in accordance with our microarray results (Figure 2E). As interferons are secreted cytokines, we sought to antagonize the interferon effect by administering antibodies against interferons α, β and γ. To that end, we initiated an interferon response by co-cultivating CAFs and carcinoma cells, leading to the elevation of IFN targets MX1 and STAT1. This elevation was exclusively abolished by the addition of anti-Interferon β antibody, and not in the presence of anti-Interferon α or γ antibodies (Figure 2F). To Verify IFN activation in HK3-T cells, we subjected these cells to conditioned media of HK3-T or that of HK3-T cultured with H1299^175^. We then measured the expression of several IFN activated proteins. Upon exposure to conditioned media from the co-culture, total STAT1 levels were not changed, however pSTAT1 and STAT2 levels were elevated (Figure 2G). To exclude the possibility of IFN activation as a result of Apoptosis/Cell death pathways, we repeated the experimental setup described in Figure 2G. Both HK3-T cells that were subjected to HK3-T Conditioned media and the ones that were subjected to cancer cells and HK3-T media, appeared viable (Figure 2H, upper panel). Accordingly, both cultures showed high viability rate (∼95%) corresponding to their PI negative populations (Figure 2H, lower panel).

### Mutant p53-bearing cells moderate CAFs-mediated interferon response

**Figure 3:**
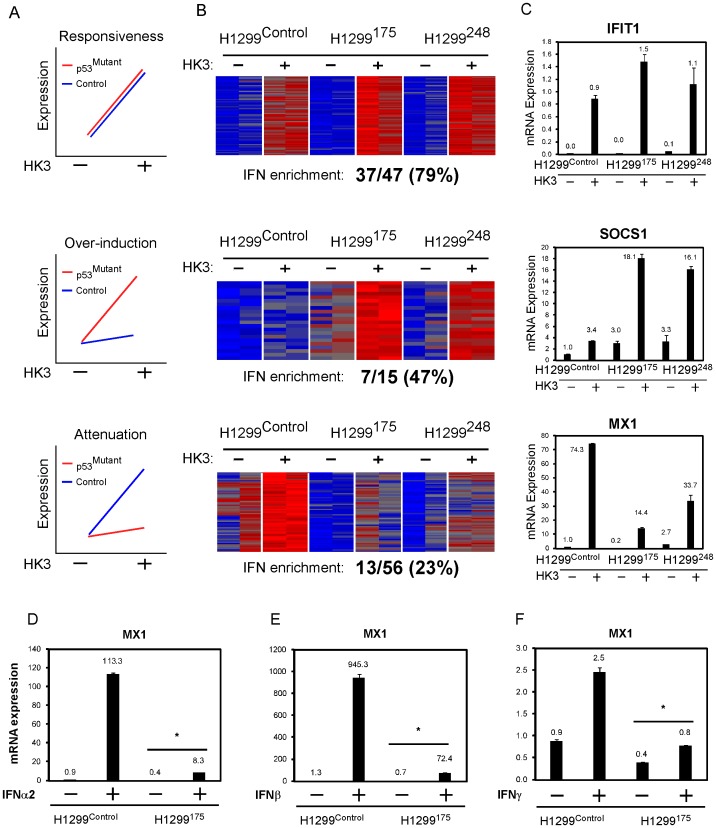
Expression patterns of H1299 in response to co-cultivation with CAFs. (A) Three principal expression patterns of H1299 cultivated with HK3-T. (B) A heat-map depicting genes that exhibit Pearson correlation of at least 0.9 to representative bait(s) from each pattern shown in A. Beneath is the percentage of IFN targets in each list based on the ‘Interferome database’ [30]. (C) QRT-PCR analysis of a representative gene from each expression pattern. (D) The H1299 panel was treated with the designated IFNs for 24 h. Shown is a QRT-PCR analysis of MX1 expression.

**Figure 4:**
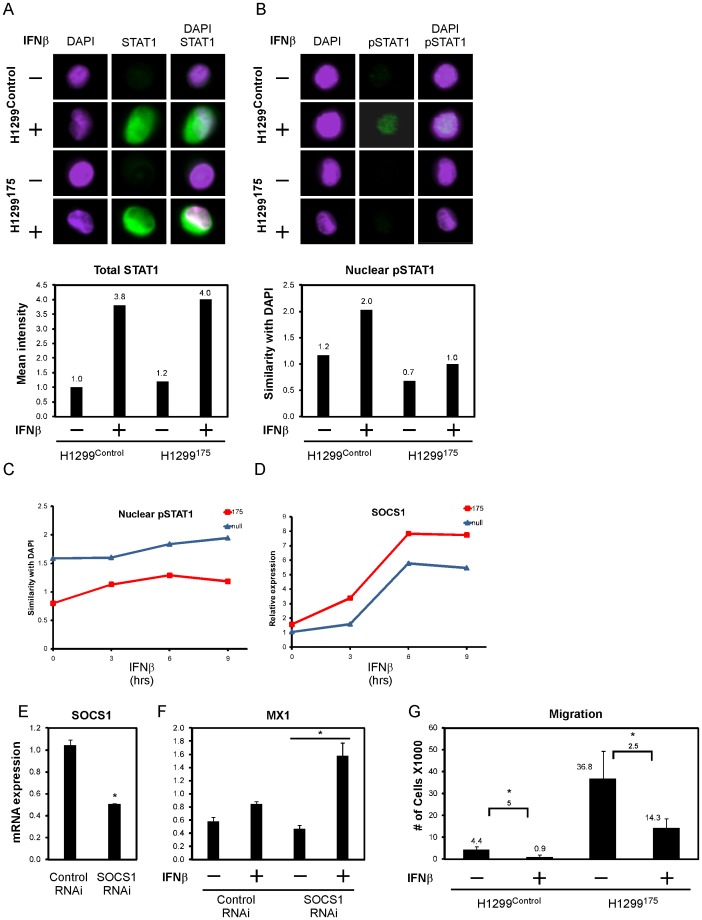
Mutant p53 counteracts IFNβ by SOCS1-mediated attenuation of STAT1 phosphorylation. (A) Cells were treated with IFNβ for 16 h, fixed and sorted by an “Image stream” FACS. The upper panel depicts representative images from each condition and the graph represents the mean pixel intensity of STAT1 positive cells for the entire population. (B) Same as in A, here the graph shows the similarity between p-STAT and DAPI staining, thereby quantifying both the expression and localization of pSTAT1. (C) Cells were treated with IFNβ for the designated durations, shown is a graph depicting nuclear p-STAT1. (D) The cells were also collected for RNA analysis and a QRT-PCR for SOCS1 expression was performed. (E) H1299^175^ cells were introduced with RNAi against LacZ as a control or against SOCS1. p = 0.002 (F) Cells were then treated with IFNβ for 24 h. Shown is a QRT-PCR analysis of MX1 expression. p<0.05. (G) Cells were seeded in trans-wells in serum-free media and treated with IFNβ for 24 h. Migrating cells were collected and counted.

In order to investigate the effect of mutant p53 in cancer cells on the surrounding fibroblasts, we analyzed the micro-array data obtained from the sorted H1299. Over-viewing differentially expressed IFN targets in H1299 that were grown alone or cultivated with CAFs, we revealed 3 major expression patterns depicted in Figure 3A: (i) responsiveness, namely both p53 null and mutant p53 bearing cells induced known interferon targets in a comparable manner, (ii) over-induction, in which IFN targets were highly induced by mutant p53 cells and (iii) attenuation, where IFN targets-induction was mitigated by mutant p53. In an effort to identify other genes that exhibit similar expression pattern, we used one gene or more from each pattern as a bait vector and searched for other genes that exhibited a Pearson correlation of at least 0.9 to the bait vector, using a custom Matlab script (Figure 3B upper panels). Next, we evaluated the frequency of IFN targets in each pattern, using the Intefreome database (Figure 3B lower panels). Expression of a representative gene from each pattern was validated by QRT-PCR (Figure 3C). Interestingly, pattern 2 (‘over-induction’) consists of two known inhibitors of the interferon signalling pathway, namely MAP3K8 and SOCS1 [32], [33]. Pattern 3 (‘attenuation’) on the other hand, consists of two known tumor inhibitors – NMI and MX1 [34], [35]. To examine whether the effect of mutant p53 on IFN pathway is a general phenomenon, we analyzed this effect in the lung cancer cell lines A549, and in SKBR3 breast cancer cells. MX1 exhibited the same expression patterns in these cell lines (Figure S3A–B), indicating that mutant p53 averts IFNs pathways at large. Notably, introducing the H1299 panel with recombinant IFNs α, β and γ, yielded similar expression patterns of MX1 (Figure 3D–F). The observation that mutant p53 had a similar effect on MX1 expression upon administration of all the IFNs suggests that mutant p53 exerts its effect on IFNs downstream targets rather than interfering with IFN itself or with its up-stream effectors. All IFNs pathways converge into the JAK1-mediated phosphorylation of STAT1, suggesting that the JAK/STAT components are affected by mutant p53. To test whether mutant p53 hinders the expression and phosphorylation of STAT1, H1299 were treated with IFNβ and stained with antibodies against p53, STAT1 and phospho-STAT1 (pSTAT1). Cells were then fixed and analyzed with the Image stream FACS sorter which photographs each individual sorted cell, thereby allowing a thorough investigation of a plethora of parameters, such as sub-cellular localization of proteins for the entire cell population. Total STAT1 levels rose following IFNβ administration, however without an apparent difference between the control and H1299^175^ (Figure 4A). Strikingly, pSTAT1 was exclusively present in the nuclei of the p53 null cells following 16 h of IFNβ treatment (Figure 4B). The same experimental setup was used with shorter time laps and revealed a continuous lower pSTAT1 levels in H1299^175^ (Figure 4C). JAK1 which phosphporylates STAT1 is known to be inhibited by SOCS1, as part of the interferon negative feedback loop. As SOCS1 belongs to the ‘over-induction’ pattern exhibited by the mutant p53 cells, we measured its expression levels as well. SOCS1 exhibited a mirror image of pSTAT1, namely was elevated in H1299^175^ during IFNβ treatment (Figure 4D). To test whether SOCS1 mediates the inhibiting effect of mutant p53, we knocked down SOCS1 expression in H1299^175^ (Figure 4E). The cells were then exposed to IFNβ treatment and indeed the expression of IFNβ targets MX1 and CXCL11 was regained (Figure 4F and Figure S4. respectively). Mutant p53 is known to facilitate invasion and migration either by promoting EMT [36], [37] or by negating p63 inhibition on invasion-promoting pathways [38]. In addition, IFNs have been reported to repress invasion of cancer cells via MX1 [35].We therefore measured the effect of IFNβ on the migratory capacity of cancer cells. First, H1299^175^ indeed proved to migrate more effectively than their p53 depleted counterparts (Figure 4G). Moreover, the ability of the latter to migrate was nullified in the presence of IFNβ. Notably, H1299^175^ migratory ability was reduced upon IFNβ treatment, however to a lesser extent. In sum, mutant p53 is able to moderate IFNβ response by over activating SOCS1 and reducing the levels of pSTAT1, thus reducing the inhibiting effect of IFNβ on cell migration.

### IFNβ attenuates mutant p53 levels through inhibition of its mRNA stabilizer, WIG1

**Figure 5:**
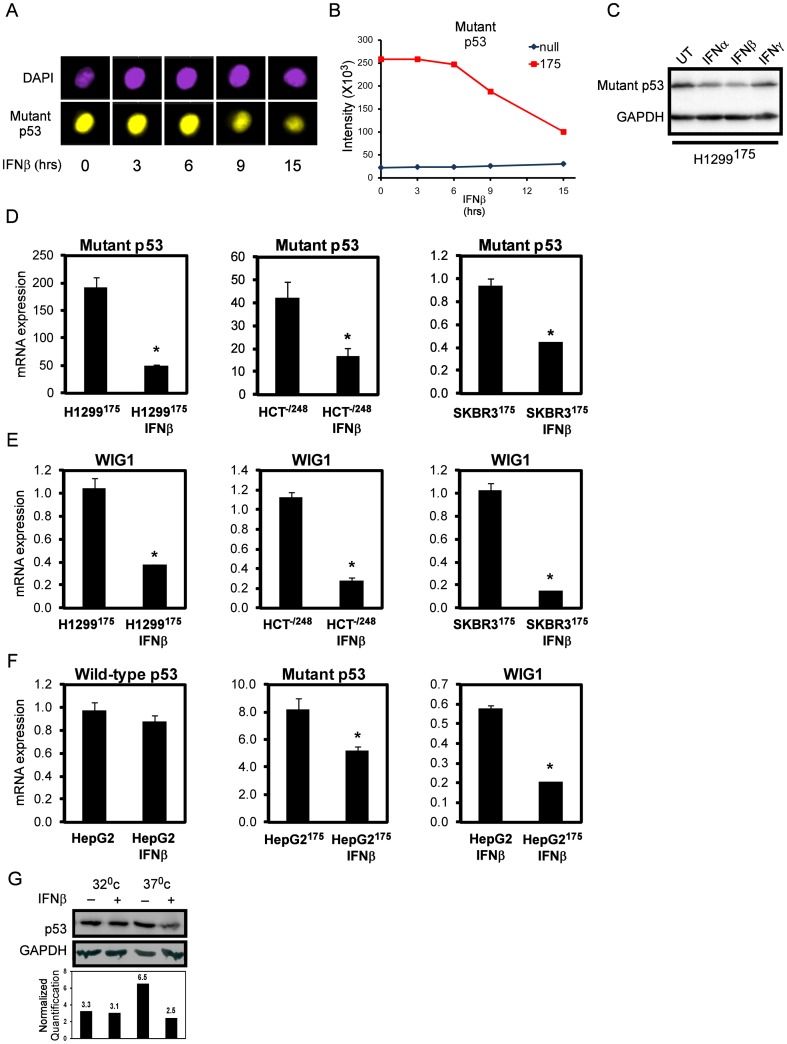
IFNβ reduces mutant p53 mRNA levels by inhibiting its RNA stabiliser WIG1 (ZMAT3). Cells were treated with IFNβ for the designated time points, fixed and sorted by an “Image stream” FACS. (A) Representative images from each condition and (B) a graph representing the mean pixel intensity of mutant p53 positive cells for the entire population. (C) Cells were treated with the designated IFNs for 24 h and mutant p53 and GAPDH levels were measured by western blot. Cells were treated with IFNβ for 24 h and mutant p53 (D) and WIG1 (E) RNA levels were determined by QRT-PCR. * P<0.05. (F) HepG2 cells were introduced with mut175 plasmid and treated with IFNβ. RNA levels were determined by QRT-PCR. * P<0.05. (G) H1299 cell harbouring a TS form of mutant p53 (Wild type form at 32°C and mutant form at 37°C) were treated with IFNβ for 30 h. p53 and GAPDH levels were measured by western blot, shown in the lower panel a is normalized quantification of the bands.

During the former set of experiments we came across an interesting phenomenon in which mutant p53 protein levels dramatically declined after 9 hours of IFNβ exposure (Figure 5A and B, Image stream analysis). To verify this finding, we administered all three IFNs for 24 hours and performed western blotting. Indeed, mutant p53 protein levels declined following IFNα, β and γ treatment. QRT-PCR analysis revealed that mutant p53 RNA levels were reduced as well (Figure 5D, left hand side). Since mutant p53 is expressed under the control of a viral promoter in our system, we wished to exclude the possibility that this observation stems from the anti-viral related effect of IFNs. For that purpose, we utilized two cell lines, which harbor endogenous p53 mutants, namely the HCT ^−/248^ knock-in cell line and SKBR3 cells which express endogenous p53^R175H^. Notably, both cell lines exhibited a significant reduction in mutant p53 RNA levels upon IFNβ treatment (Figure 5D, right hand side). These observations suggested that IFNβ compromises mutant p53 RNA stability.

Wild type p53 is instrumental for cell fate decisions and is therefore subjected to several tiers of control. One mode of regulation is exerted on its mRNA molecule in terms of stability and translation. WIG1 is a zinc finger protein capable of binding a U-rich element in the 3′ region of p53 mRNA, thereby inhibiting its de-adenylation and increasing its stability [39]. As both wild type and mutant p53 mRNAs have identical 3′ sequences, mutant p53 benefits from WIG1 activity and indeed Vilborg et. al. have shown that mutant p53 levels decrease following WIG1 knockdown [40]. We therefore decided to examine whether WIG1 is affected by IFNβ. Indeed, WIG1 levels decreased upon IFNβ treatment in all tested cell lines (Figure 5E). These observations were not restricted to human cells as WIG1 down-regulation was also evident in mouse B-cells treated with IFNβ (Figure S5, data retrieved from a micro-array by [41]).

Several studies documented a positive interaction between IFNβ and wild type p53 [22], [23], [24], [42], thus WIG1-mediated repression of wild type p53 by IFNβ seems to be counter-intuitive. While WIG1 is a *bona fide* target of wild type p53 [43], [44], mutant p53 seems to exert a dominant negative effect over its expression [45]. We therefore hypothesized that a differential effect of IFNβ on mutant and wild type p53 RNA levels might be achieved by the wild type specific targeting of WIG1. As illustrated in Figure 5F, HepG2 cells expressing either wild type or mutant p53 were subjected to IFNβ and only mutant p53 levels were reduced. Notably, WIG1 levels were significantly lower in the mutant p53 expressing cells. Thus, only wild type p53 can bypass the attenuating effect of IFNβ on WIG1 expression and maintain intact stable pool of mRNA. Currently, it is still unclear whether WIG1 inhibits or promotes tumor progression [46]. To substantiate the differential effect of IFNβ on wild type vs. mutant p53, we used H1299 harboring a mutated Temperature Sensitive (TS) form of p53. At 37°C, this form is at a mutated conformational state, whereas at 32°C it shifts to a wild type conformation. This is a common system for comparing wild type and mutant p53, on an isogenic background [47]. Indeed as shown in figure 5E by western blot, at 32°C, IFNβ had no effect on p53 levels, while at 37°C it reduced mutant p53 protein levels by more than half.

The above findings suggest that it might involve a mutant p53-dependent mechanism.

## Discussion

**Figure 6:**
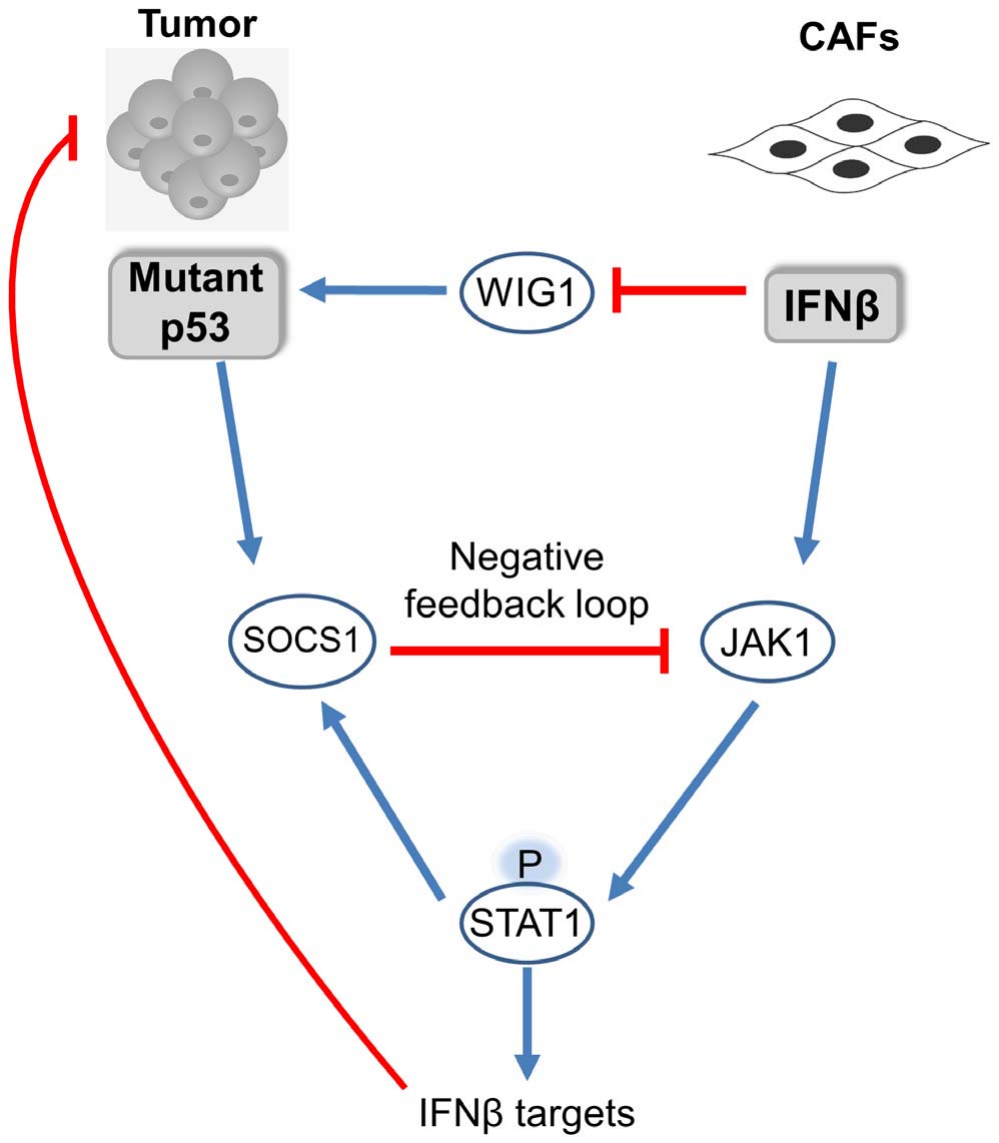
A schematic representation of the interaction between mutant p53 and the IFNβ pathway. Blue arrows denote positive effect while red arrows denote negative effect. Upon encounter with cancer cell CAFs activate the IFNβ pathway which limits cancer cells' migration. When mutant p53 is present in the cancer cells, this pathway is moderated via SOCS1 mediated inhibition of STAT1 phosphorylation. IFNβ is able to reduce mutant p53 RNA levels by attenuating the expression of mutant p53 RNA stabilizer WIG1.

The tumor microenvironment and its effect on cancer cells is one of the leading paradigms in cancer research. CAFs, which are often abundant in the tumor stromal milieu, have been reported to mediate the tumor promoting effect of the stroma to various extents. In our work we set out to characterize CAFs response to cancer cells expressing mutant p53 and vice versa. As summarized in Figure 6, we found that CAFs secrete IFNβ in the presence of cancer cells, which attenuates the migration of the latter. Mutant p53 moderates the response to IFNβ in the cancer cells via SOCS1-mediated inhibition of STAT1 phosphorylation. IFNβ on the other hand, reduces mutant p53 RNA levels by restricting its RNA stabilizer, WIG1. In light of our findings several intriguing notions which come to mind and are described below.

### IFNβ as an alternative CAFs induced pro-inflammatory pathway

Recently, several reports have documented a link between CAFs and cancer-related inflammation. IL1α/β and TNFα secreted by the tumor cells are common paracrine activators of CAFs induced inflammation in a variety of cancers and experimental models [48], [49], [50], [51], [52]. Following this activation, CAFs initiate a pro-inflammatory response, which may affect tumor growth in a direct manner or induce inflammation via recruitment of the immune system [48], [49], [53]. Nuclear factor-κB (NFκB) seems to serve as hub, which orchestrates CAFs mediated pro-inflammatory response [49], [54]. During our preliminary experiments, the NFκB and its downstream components were measured. However, this pathway seems to remain unresponsive in the co-culture setup. CAFs induced IFNβ response combined with its known ability to recruit the immune system, in an NFκB depleted background - allude to the possibility that IFNβ might provide with an alternative pro-inflammatory pathway. As this phenomenon was evident with other pairs of CAFs and cancer cells, it could be surmised that IFNβ and the NFκB pathways act in a mutually exclusive fashion.

### IFNβ and mutant p53 – the clinical standpoint

The use of mutant p53 in prognosis and as a predictor of survival and clinical outcome has been a matter of debate for many years, mainly due to technical issues. However, the general trend links mutant p53 with poor survival in several cancers [55]. In addition, individuals that carry p53 mutations in their germ-line are associated with the Li-Fraumeni syndrome manifested by the early onset of several cancers. Indeed, drugs such as low-molecular weight compounds and short peptides were developed, aimed at restoring p53 wild-type activity, by shifting the wild-type and mutant equilibrium towards the wild-type conformation [56]. Such are the compound MIRA-1 [57] and the short peptides CDB3 and CP-31398 [58], [59]. Several recombinant IFNβs have been approved for the treatment of multiple sclerosis (FDA, 2012), and there are few ongoing clinical trials utilizing IFNβ as an anti-cancer therapy (ClinicalTrials.Gov, 2012). Recent years have underscored the clinical need for tailoring personalized anti-cancer drugs to the proper recipients based on the genomic landscape of their specific tumors. As mutant p53 detection is considered standard protocol in many oncological departments, coupled with the fact that there are four FDA approved IFNβs, we propose, given our findings, to direct IFNβ treatment to patients carrying p53 mutations, thus increasing their survival and improving their prognosis. Moreover treating Li-Fraumeni patients carrying p53 mutations with regular dosage of IFNβ might prove to have a long lasting preventative effect against cancer in those patients. IFNα is another FDA approved anti-cancer drug in a variety of tumor types, either as a stand-alone treatment, as an adjuvant or in combination with other drugs [60]. It is tempting to speculate that stratifying patients according to their mutant p53 type will aid in improving IFNα performance. The fact that WIG1 seems to be inhibited by IFNs alludes to the possibility that more WIG1 tumor-promoting targets other than mutant p53 might be reduced by IFNs. For example, N-Myc has been recently reported to be regulated by WIG1 [40]. In addition ΔN73, a p53 family member which bears sequence resemblance to p53 [61] and is considered to be oncogenic, was reported to be down regulated on the RNA level by both IFNα and IFNβ [23], [62], perhaps due to WIG1 inhibition. Future efforts should be aimed at characterizing WIG1 targeted tumor promoters, keeping in mind that these data could assist in tailoring IFNs treatment to the right patients.

## Materials and Methods

### Cell lines

HK3-T lung CAFs establishment was previously described [10]. Cells were cultured in a humidified incubator at 37°C and 5% CO_2_. CAFs and HepG2 were grown in MEM, H1299 and SKBR-3 (Purchased from ATCC) in RPMI, A549 in DMEM and HCT116 in McCoy's Media supplemented with 10% FCS and Pen/Strep solution (Biological industries, Beit-Haemek, Israel).

### Western blot Analysis

Total cell extracts were fractionated by gel electrophoresis; proteins were transferred to nitrocellulose membranes, and immunoblotted using the designated antibodies: anti-GAPDH mab374, (Chemicon, Billerica, MA) anti-p53 DO1, mouse p53 Ab C-2524S (Cell Signaling) and anti-STAT2 DB028 (c20) Polyclonal (Delta Biolabs). The protein-antibody complexes were detected by horseradish peroxidase-conjugated secondary antibodies followed by the enhanced SuperSignal west pico chemiluminescent substrate (Thermo scientific, IL, USA).

### Isolation of Total RNA and Quantitative Real-Time PCR (QRT-PCR)

Total RNA was isolated using the NucleoSpin RNA II kit (Macherey-Nagel), according to the manufacturer's protocol. A 2 ug aliquot was reverse transcribed using MMLV-RT (Bio-RT) and random hexamer primers. QRT-PCR was performed on an ABI 7300 instrument (Applied Biosystems) using Platinum SYBR Green and qPCR SuperMix (Invitrogen). Primers sequences are listed in Table S1. Data analysis was performed according to the ΔΔCt method using HPRT as the endogenous control. The results are presented as a mean±S.D. of two or three duplicate runs from a representative experiment.

### Image Stream FACS

Cells were collected, trypsinized and supplemented with 5 mM EDTA, washed and reconstituted in 70% ETOH – HBSS and incubated for 1 hr in −20°c. Cells were then blocked with 3%BSA-PBS and supplemented with the designated antibodies: anti-p53 DO-1, anti-STAT1 p91, C-24:sc-456 and anti-pSTAT1 Tyr 701:sc-7988 (Santa-cruz). Cells were then washed and supplemented with fluorescent antibodies (DAPI, cy3 and cy5). After washing, cells were centrifuged and reconstituted in 100 µl, sorted and analyzed. As controls, each dye was measured alone and its penetration to other channels was deducted from all other channels. For nuclear localization, the similarity between the mean intensity of DAPI and the desired protein was calculated.

### Interferons treatment

Recombinant human Interferon α (#300-02-AB), β (#300-02BC-100), and γ (#300-02-100), and their corresponding antibodies: α (500-P32A), β (500-P32B), and γ (500-P32), were purchased from Peprotech, Israel. IFNs concentrations used in this study were as follows: IFNα - 1000 units/ml, IFNβ - 1 nM, and IFNγ 10 ng/ml.

### SOCS1 knockdown

Cells (5×10^4^) were seeded in a 6 cm plate and were treated with siRNA against either SOCS1 or LacZ as a control according to the manufacturer protocol (Thermo scientific) for 48 hrs.

### Statistical analysis

Unless stated otherwise, an unpaired one-tailed student t-test was performed. * denotes at least p<0.05.

### cDNA Microarray

Total RNA was extracted using Tri-Reagent (MRC Inc.) according to manufacturer's protocol, and sent to the MicroArray unit (Weizmann institute of science, Rehovot, Israel). Agilent chips (Human 8X60K) were used as a platform for RNA loading. Each sample expression was compared to a common reference sample comprised of an equal amount of RNA from all samples. The limma package [63] was used for microarray processing. Background was corrected using the function backgroundCorrect and normalization within and between arrays was performed using the functions normalizeWithinArrays and normalizeBetweenArrays, respectively. Spots with the same probes were averaged. Analysis of variance (ANOVA) including contrasts was applied to the data set using Partek Genomic Suite 6.5 (Inc. St. Charles, MO).

The microarray data from this publication have been submitted to the Gene Expression Omnibus (GEO) database and assigned the identifier accession GSE41477.

## Supporting Information

Figure S1 **(Related to** **Figure 1** **) The effect of the sorting procedure on stromal and cancer cells.** QRT-PCR was performed and relative expression of GFP and dsRed following the second sort is shown (A). GFP labeled cells are written in green and dsRed labeled cells are written in red. Parentheses denote the adjacent population that was not collected. (B)The designated cells were sorted under the same conditions described above. Cell pellets were collected prior and post sorting and the mRNA levels of the designated stress related genes were measured by QRT-PCR.
(TIF)

Figure S2 **(Related to** **Figure 2** **) The interferon pathway is up regulated in some but not all pairs of CAFs and lung carcinoma cells.** (A–C) CAFs were co-cultured with the designated carcinoma cells lines and with each other. Shown is a QRT-PCR for MX1. * denotes P<0.05.
(TIF)

Figure S3 **(Related to** **Figure 3** **) MX1 is attenuated by mutant p53 following IFNβ treatment.** (A) A549 were introduced with an empty vector or mutant p53 (R175H) vector, and treated with IFNβ for 24 h. Shown is a QRT-PCR for MX1. (B) SKBR3 which express endogenous mutant p53 (R175H) were introduced with shRNA vector targeting non-human sequence and mutant p53 and treated with IFNβ for 24 h. Shown is a QRT-PCR for MX1.
(TIF)

Figure S4 **(Related to** **Figure 4** **) SOCS1 mediates mutant p53 attenuating effect on CXCL11 following IFNβ treatment.** (A) H1299^175^ cells were introduced with RNAi against Lacz as a control or SOCS1and treated with IFNβ for 24 h. Shown is QRT-PCR for SOCS1 and MX1 expression.
(TIF)

Figure S5 **(Related to** **Figure 5** **) IFNβ attenuates WIG1 in mouse B-cells.** (A) In a microarray retrieved from [41] mouse B cells were treated with IFNβ. Shown is the average WIG1 expression of four replicates. p = 0.003.
(TIF)

Table S1 **QRT-PCR primers.**
(DOCX)
