# Supplementary figures and images for: Mutant p53 Attenuates the Anti-Tumorigenic Activity of Fibroblasts-Secreted Interferon Beta
Source: PLoS One. 2013 Apr 22;8(4):e61353. doi: 10.1371/journal.pone.0061353 (PMC3632588; doi:10.1371/journal.pone.0061353)

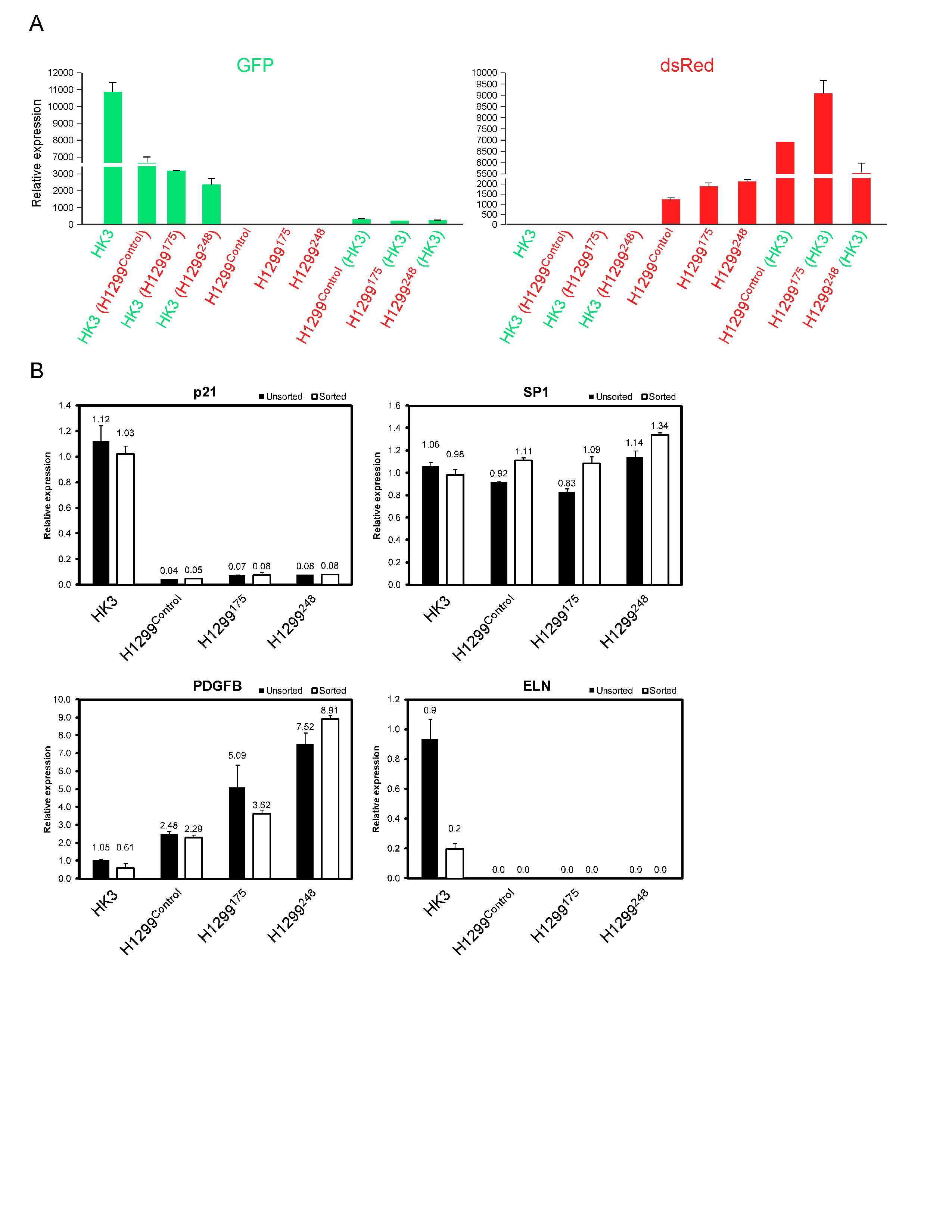

Supplement: Figure S1 — (Related to Figure 1 ) The effect of the sorting procedure on stromal and cancer cells. QRT-PCR was performed and relative expression of GFP and dsRed following the second sort is shown (A). GFP labeled cells are written in green and dsRed labeled cells are written in red. Parentheses denote the adjacent population that was not collected. (B)The designated cells were sorted under the same conditions described above. Cell pellets were collected prior and post sorting and the mRNA levels of the designated stress related genes were measured by QRT-PCR. (TIF) [file pone.0061353.s001.tif]
