# Supplementary material for: Mutant p53 Attenuates the Anti-Tumorigenic Activity of Fibroblasts-Secreted Interferon Beta
Source: PLoS One. 2013 Apr 22;8(4):e61353. doi: 10.1371/journal.pone.0061353 (PMC3632588; doi:10.1371/journal.pone.0061353)
Supplement: Table S1 — QRT-PCR primers. (DOCX) [file pone.0061353.s006.docx]

**Supporting Information Table 1 - QRT-PCR primers**

| **Symbol** | **Forward primer** | **Reverse primer** |
| --- | --- | --- |
| IFNα* | CCCTCTCTTTATCAACAAACTTGC | TTGTTTTCATGTTGGACCAGA |
| IFNβ* | CGACACTGTTCGTGTTGTCA | GAAGCACAACAGGAGAGCAA |
| MX1 | GTTTCCGAAGTGGACATCGCA | CCATTCAGTAATAGAGGGTGGGA |
| STAT1 | ATGTCTCAGTGGTACGAACTTCA | TGCTCCAGGAATTTTGAGTCAAG |
| SOCS1* | CCCCTGGTTGTTGTAGCAG | GTAGGAGGTGCGAGTTCAGG |
| p53 | CCCAAGCAATGGATGATTTGA | GGCATTCTGGGAGCTTCATCT |
| WIG1 | CGGCAGAGAATTCCACGTGAT | ATCTCTTCGCCAGCTCCAACA |
| GFP | GGGCACAAGCTGGAGTACAAC | CACCTTGATGCCGTTCTTCTG |
| dsRed | GCTCCTCCAAGAACGTCATC | GGGTGCTTCACGTACACCTT |
| SP1 | AAACGTACACACACAGGTGAGAAGA | CTCCTCATGAAGCGCTTAGGA |
| p21 | GGCAGACCAGCATGACAGATT | GCGGATTAGGGCTTCCTCT |
| ELN | GCAGGAGTTAAGCCCAAGG | TGTAGGGCAGTCCATAGCCA |
| PDGFB | TTAAGAAGGCCACGGTGACG | TCTCACACTTGCATGCCAGG |
| CXCL11 | GACGCTGTCTTTGCATAGGC | GGATTTAGGCATCGTTGTCCTTT |
| IFIT1 | AAGCTTTCAAATCCCTTCCGC | GCCTTGGCCCGTTCATAATT |

* Roche taqman primers
